# Eighteen year weight trajectories and metabolic markers of diabetes in modernising China

**DOI:** 10.1007/s00125-014-3284-y

**Published:** 2014-06-03

**Authors:** Penny Gordon-Larsen, Elizabeth Koehler, Annie Green Howard, Lauren Paynter, Amanda L. Thompson, Linda S. Adair, Elizabeth J. Mayer-Davis, Bing Zhang, Barry M. Popkin, Amy H. Herring

**Affiliations:** 1 Department of Nutrition, Gillings School of Global Public Health, University of North Carolina, Chapel Hill, NC USA; 2 Carolina Population Center, University of North Carolina at Chapel Hill, University Square, 123 West Franklin Street, Chapel Hill, NC 27516-3997 USA; 3 Department of Biostatistics, Gillings School of Global Public Health, University of North Carolina, Chapel Hill, NC USA; 4 Department of Anthropology, University of North Carolina, Chapel Hill, NC USA; 5 Department of Medicine, University of North Carolina, Chapel Hill, NC USA; 6 Institute of Nutrition and Foods Safety, Chinese Centers for Disease Control, Beijing, People’s Republic of China

**Keywords:** Adult, China, Fasting glucose, Insulin resistance, Latent class trajectory analysis

## Abstract

**Aims/Hypothesis:**

Although obesity is a major risk factor for diabetes, little is known about weight gain trajectories across adulthood, and whether they are differentially associated with metabolic markers of diabetes.

**Methods:**

We used fasting blood samples and longitudinal weight data for 5,436 adults (5,734 observations, aged 18–66 years) from the China Health and Nutrition Survey (1991–2009). Using latent class trajectory analysis, we identified different weight gain trajectories in six age and sex strata, and used multivariable general linear mixed effects models to assess elevated metabolic markers of diabetes (fasting glucose, HbA_1c_, HOMA-IR, insulin) across weight trajectory classes. Models were fitted within age and sex strata, and controlled for baseline weight (or baseline weight by weight trajectory interaction terms), height, and smoking habit, with random intercepts to control for community-level correlations.

**Results:**

Compared with weight gain, classes with weight maintenance, weight loss, or a switch from weight gain to loss had lower values for metabolic markers of diabetes. These associations were stronger among younger women (aged 18–29 and 30–39 years) and men (18–29 years) than in older (40–66 years) men and women. An exception was HOMA-IR, which showed class differences across all ages (at least *p* < 0.004).

**Conclusion:**

Trajectory analysis identified heterogeneity in adult weight gain associated with diabetes-related metabolic markers, independent of baseline weight. Our findings suggest that variation in metabolic markers of diabetes across patterns of weight gain is masked by a homogeneous classification of weight gain.

**Electronic supplementary material:**

The online version of this article (doi:10.1007/s00125-014-3284-y) contains peer-reviewed but unedited supplementary material, which is available to authorised users.

## Introduction

Although obesity is a major risk factor for diabetes [1], the long-term patterns of adult weight gain associated with diabetes and insulin resistance are not well understood. Much previous research has used simple measures of weight change, typically assuming a population average trajectory [2–5]. Some recent reports have characterised weight change using methods to derive patterns, such as principal components [6, 7], to examine diabetes risk. However, more complex methods can identify distinct groups with similar underlying weight trajectories that differ in their functional form [8–10]. While such methods have been used to classify trajectories of weight change [11–14], they have not been widely used to examine differential metabolic markers of diabetes as a function of different weight trajectory patterns across adulthood.

China presents a unique model for weight change, having recently undergone transition from a history of undernutrition to a rapid increase in obesity [15, 16]. In addition, the considerable geographic and temporal heterogeneity in the timing of the transition from underweight to overweight across China provides sufficient variation in the shape of weight trajectories to investigate a potential differential association with diabetes markers. The incidence of obesity-related noncommunicable diseases, such as diabetes, has more than doubled over the past two decades from approximately 3% in 1994 to 7–10% in 2008 [17, 18]. Such diseases are now the leading causes of morbidity, disability and mortality in China [15, 19].

This study uses longitudinal weight data for 5,436 individuals (25,734 observations), along with markers of diabetes (fasting glucose, HbA_1c_, insulin and insulin resistance [HOMA-IR]) obtained in 2009. Using latent class trajectory modelling to characterise weight trajectories over 18 years, we examine longitudinal patterns of weight gain to determine whether subgroups of individuals with different weight trajectories show variations in glucose, HbA_1c_, HOMA-IR and insulin levels. We tested the hypothesis that, after controlling for initial weight, a large weight gain over 18 years is associated with higher levels of glucose, HbA_1c_, HOMA-IR and insulin compared with stable weight or a smaller weight gain over the same time period.

## Methods

### The China Health and Nutrition Survey

The China Health and Nutrition Survey (CHNS) collected health data in 228 communities (nine diverse provinces: Guangxi, Guizhou, Heilongjiang, Henan, Hubei, Hunan, Jiangsu, Liaoning and Shandong) throughout China in seven survey rounds from 1991 to 2009 (1991, 1993, 1997, 2000, 2004, 2006 and 2009). The 2009 survey was the first to collect fasting blood samples. Using a multistage, random cluster design, a stratified probability sample was used to select counties and cities stratified by income and urbanicity using State Statistical Office definitions [20]. Communities and households were then randomly selected from these strata. The CHNS cohort initially mirrored national age–sex–education profiles [21–23], and by 2011 the provinces in the CHNS sample constituted 47% of the Chinese population (according to the 2010 census). Survey procedures have been described elsewhere [24]. The study was approved by the Institutional Review Board at the University of North Carolina at Chapel Hill, the China–Japan Friendship Hospital, the Ministry of Health and China, and the Institute of Nutrition and Food Safety, China Centers for Disease Control. Participants gave informed consent.

### Study population

The present analysis limited eligibility to adults aged 18 years at study entry to 66 years at the 2009 examination (to avoid age-related reductions in weight caused by sarcopenia [25]), with biomarker data (*n* = 8,149). Additional inclusion criteria were anthropometric measures from at least two surveys to derive weight trajectories (*n* = 6,470), fasting blood collection, and not pregnant at 2009 (*n* = 5,436). The number of visits providing anthropometry measures ranged from two to seven measurement occasions (two visits, *n* = 666; three visits, *n* = 885; four visits, *n* = 842; five visits, *n* = 1,059; six visits, *n* = 804; seven visits, *n* = 1,180; median = five visits; total *n* = 5,436 participants across 25,734 observations).

### Measures

#### Diabetes indicators

Following overnight fasting, a 12 ml blood sample was collected by venipuncture. Whole blood was immediately centrifuged and serum was tested for glucose using a glucose oxidase phenol 4-aminoantipyrine peroxidase kit (Randox, Crumlin, UK) and a Hitachi 7600 analyzer (Hitachi; Tokyo, Japan). Serum insulin was tested using radioimmunology assay kit (North Institute of Biological Technology; Beijing, China) using a XH-6020 gamma counter (North Institute of Biological Technology). Whole blood HbA_1c_ high performance liquid chromatography analysis (model HLC-723 G7; Tosoh Corporation, Tokyo, Japan) generated continuous outcomes for fasting glucose, HbA_1c_, insulin and natural log transformed HOMA-IR, calculated as (insulin × glucose/6.945 pmol/l) [26, 27].

#### Anthropometry

At each visit, the height of participants was measured without shoes to the nearest 0.2 cm using a portable SECA stadiometer (SECA; Hamburg, Germany). Weight was measured without shoes and in light clothing to the nearest 0.1 kg using a calibrated beam scale.

#### Weight trajectories: latent class trajectory modelling

We used latent class trajectory modelling (LCTA) to identify weight gain trajectories using the SAS version 9.2 (SAS Institute, Cary, NC) TRAJ procedure with a censored normal model [28, 29]. LCTA has only recently been used to identify otherwise unresolved trajectory classes in epidemiological data [30]. Unlike traditional growth curve analysis, which assumes that individuals vary around a single mean growth curve, LCTA has the advantage of classifying individuals into distinct groups with similar underlying trajectories [8–10]. The differential age-related patterns of weight gain in China over the past two decades provided the rationale for examining trajectories within age groups. We modelled weight change in six sex-by-baseline age subgroups (male, female: 18 to 29 years, 30 to 39 years, and 40 to 66 years), allowing for a variety of different order polynomials. We used statistically rigorous criteria to determine best fit via: (1) model selection using lowest Bayesian information criterion, a well-accepted model comparison metric often used for latent class models [31]; and (2) inclusion of at least 2% of the sample population within each trajectory class. Although models might differ across age groups according to different order polynomials, trajectories were assumed to follow the same order polynomial within each age group. Once weight gain trajectories were determined, a nominal categorical variable was created to describe the trajectory membership of each individual, which was then used in central analyses.

#### Demographic and behavioural variables

Ever smoking and number of cigarettes per day were reported at each exam. Urbanicity was defined using a multidimensional 12 component urbanisation index capturing community-level physical, social, cultural and economic environments, and represented the heterogeneity otherwise missed in an urban vs rural measure based only on population density [32]. Household income was reported in Yuan.

### Statistical analyses

After class identification, analyses used R (version 2.13; R Development Core Team, Vienna, Austria) [33]. Demographic variables and continuous and categorical diabetes-related outcomes were summarised across age and sex strata as percentages (categorical variables), median, and 25th and 75th percentiles (continuous variables). Continuous diabetes-related outcomes were compared across weight trajectory classes within age and sex strata using multivariable general linear mixed effects models that included covariates determined a priori to be of interest: (1) baseline weight; (2) mean adult height (averaged across all repeated measures); (3) time; (4) ever smoker status; and (5) current number of cigarettes. Random intercepts were included to account for community-level correlations. Baseline weight, height and number of cigarettes (for men only, given the low prevalence [3.3%] of female smokers) were included as cubic splines with four knots at equally spaced quartiles based on distribution. The use of flexible splines allowed us to capture a true linear trend when necessary and provided protection against possible misspecification, without imposing order on the trajectories. We accounted for the relationship between baseline weight and trajectory by including weight in the model (the combination of baseline weight and trajectory effectively describe an individual’s weight in 2009), and adjusted for height to estimate the effects of trajectories independent of height. The statistical significance of an interaction between trajectory class variables with baseline weight was set at *p* < 0.05, and results were compared with models with and without trajectories to test group differences. We systematically tested whether diabetes-related outcomes differed depending upon (1) trajectory and (2) specific class differences. Such testing did not impose order on the trajectories.

To aid the interpretation of findings, expected diabetes-related outcomes with 95% CIs are shown in all figures derived from model results set at average covariate levels for each weight trajectory class within each age–sex strata. For each model, an overall test for weight trajectories was included when testing for statistically significant differences between classes. In sensitivity testing, model fit was compared between trajectory models and a set of similarly specified models using only the most recent weight (as opposed to the weight trajectories and baseline weight). This was done to assess whether weight trajectories provided important additional information for predicting elevated values of metabolic markers of diabetes. Models were compared using Akaike information criteria.

We conducted a simulation study to identify correlations between the rank of the trajectory classes and levels of metabolic markers of diabetes. Ranks of the trajectory groups were recorded, with rank 1 being assigned to the trajectory class with the highest estimate. This process was repeated 1,000 times to determine the frequency of each ranking for each trajectory class. We also conducted a sensitivity analysis to compare results with and without the 139 individuals who reported taking diabetes medications or insulin, or who reported that they had been diagnosed with diabetes by a doctor.

## Results

### Participant characteristics

Compared with the overall eligible CHNS population (*n* = 8,149), the group included in the study (*n* = 5,436) was older, of lower weight, shorter and from less-urbanised areas, and had smoked more than the excluded group (*n* = 2,713; see Electronic supplementary material [ESM] Table 1).

Overall, the median age in 2009 was 49.0 (range 21–66) years and the median baseline weight was 56.4 kg (Table 1). There was variation across age strata in income and urbanisation, and higher levels of smoking in men.

**Table 1:** Demographic characteristics of the study population by sex and age strata

Strata	*n*	Age in 2009 (years)	Baseline weight	Average adult height	Mean BMI	Total household income	Urbanisation index (2009)	Number of cigarettes per day	Ever smoker (%, *n*)
Overall	5,436	49.0 (40.8–56.8)	56.4 (51.0–63.5)	160.9 (155.7–167.0)	22.6 (20.9–24.7)	29,117 (15,613–50,560)	61.0 (50.6–83.0)	0.0 (0.0–8.0)	31.9 (1,736)
Women									
18–29	899	39.1 (34.1–44.4)	51.5 (47.3–56.0)	157.5 (154.1–160.3)	21.7 (20.2–23.8)	32,512 (17,511–52,727)	58.4 (49.7–82.1)	0.0 (0.0–0.0)	1.0 (9)
30–39	1,054	50.2 (43.8–54.2)	53.2 (48.6–59.5)	156.3 (152.3–160.0)	22.7 (21.1–24.7)	26,993 (14,358–49,751)	63.0 (51.3–82.9)	0.0 (0.0–0.0)	2.8% (30)
40–66	889	59.7 (55.6–62.4)	55.5 (49.3–63.0)	155.5 (151.6–159.4)	23.5 (21.7–25.8)	26,222 (13,073–46,437)	66.7 (50.6–85.4)	0.0 (0.0–0.0)	7.2 (64)
Men									
18–29	904	37.9 (31.9–43.0)	59.0 (54.5–64.6)	168.1 (164.3–172.0)	21.9 (20.5–23.8)	31,892 (18,255–54,167)	59.4 (49.3–82.3)	8.0 (0.0–20.0)	61.3 (554)
30–39	838	50.4 (44.7–53.9)	61.0 (55.0–67.5)	166.9 (162.2–171.2)	22.8 (21.1–24.8)	28,961 (15,421–51,826)	63.0 (50.6–83.0)	10.0 (0.0–20.0)	63.7 (534)
40–66	852	59.5 (55.6–62.5)	61.2 (55.0–69.8)	166.0 (162.2–170.0)	22.9 (21.1–25.0)	27,654 (15,655–47,983)	61.1 (49.7–84.0)	7.0 (0.0–20.0)	64.0 (545)

Data shown as median (25th to 75th percentile), except where otherwise indicated. Statistical significance for heterogeneity for each variable by age group was set at *p* < 0.001

In general, levels of metabolic markers of diabetes were higher in older age groups. Exceptions were insulin, which was slightly lower in older men, and log HOMA-IR, which was lower in 30–39-year-old men (Table 2).

**Table 2:** Diabetes and insulin markers by sex and age strata

Strata	*n*	Glucose (mg)	HbA_1c_ (%; mmol/mol)	Insulin (μIU/ml)	Log HOMA-IR
Women					
18–29	899	88.2 (82.8–95.4)	5.3 (5.1–5.6); 34 (32.2–37.7)	9.9 (7.4–14.3)	0.79 (0.46–1.15)
30–39	1,054	91.4 (84.6–99.2)	5.5 (5.2–5.8); 36.6 (33.3–39.9)	10.2 (7.2–14.5)	0.81 (0.46–1.20)
40–66	889	93.4 (86.0–102.6)	5.6 (5.4–6.0); 37.7 (35.5–42.1)	10.8 (7.7–15.9)	0.93 (0.53–1.38)
Men					
18–29	904	88.4 (82.4–97.0)	5.4 (5.1–5.7); 35.5 (32.2–38.8)	10.7 (7.4–15.3)	0.85 (0.44–1.26)
30–39	838	92.2 (84.6–102.8)	5.5 (5.2–5.8); 36.6 (33.3–39.9)	9.4 (6.6–13.7)	0.78 (0.39–1.20)
40–66	852	93.3 (85.5–103.1)	5.6 (5.3–5.9); 37.7 (33.3–41)	9.9 (6.7–14.3)	0.84 (0.39–1.25)

Data shown as median (25th to 75th percentile). Statistical significance for heterogeneity for each variable by age group was set at *p* < 0.001

IU, international units

### Weight trajectories and metabolic markers of diabetes

**Fig. 1:**
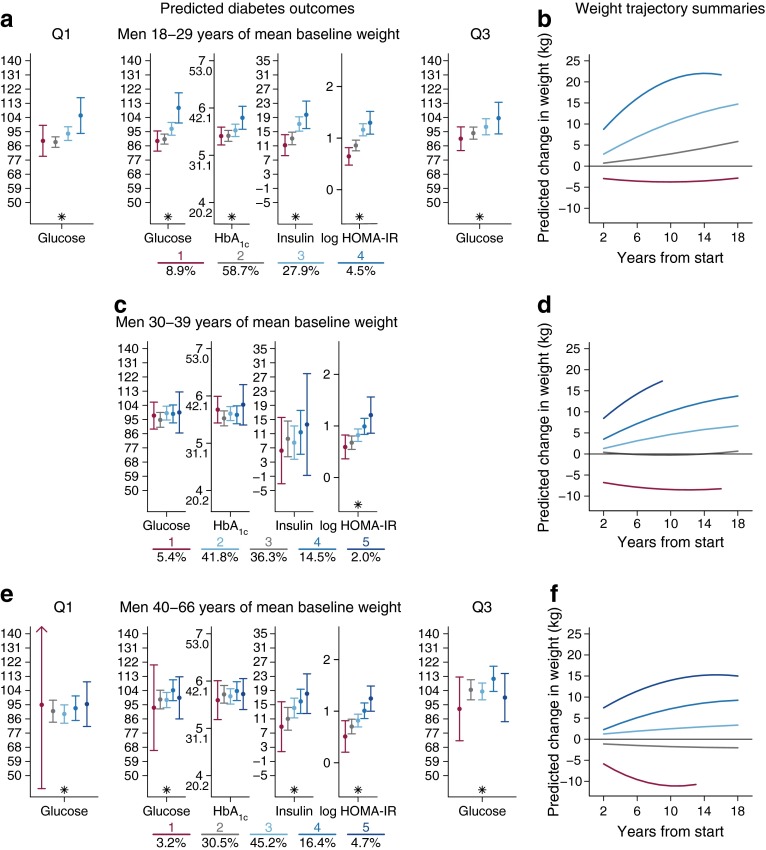
Predicted metabolic markers of diabetes by corresponding weight change trajectory summaries across three age strata in men. Predicted diabetes outcomes were generated from general linear mixed models: (**a**, **b**) 18–29 years; (**c**, **d**) 30–39 years; (**e**, **f**) 40–66 years. Data represent the expected outcomes with 95% CIs at three baseline weights: the sex-specific 25th percentile (quartile Q1), average, and 75th percentile (quartile Q3), where interaction between trajectory class and baseline weight was statistically significant (**a**, **e**), and by average baseline weight where the interaction was not statistically significant (**c**). Each *y*-axis corresponds to the outcome noted below the figure. Results are shown for never smokers with mean baseline weight (62 kg) and mean adult height (167 cm), living in an average community. The percentage sample in each class is shown below the graphs. *p < 0.05 for group differences in an overall test for weight trajectories. Weight trajectory summaries show a different colour for each weight trajectory class (**b**, **d**, **f**), with percentage of sample in each class shown below parts (**a**), (**c**) and (**e**). Shorter lines in (**b**), (**d**) and (**f**) refer to a shorter study period for individuals who entered the study in the mid-2000s

**Fig. 2:**
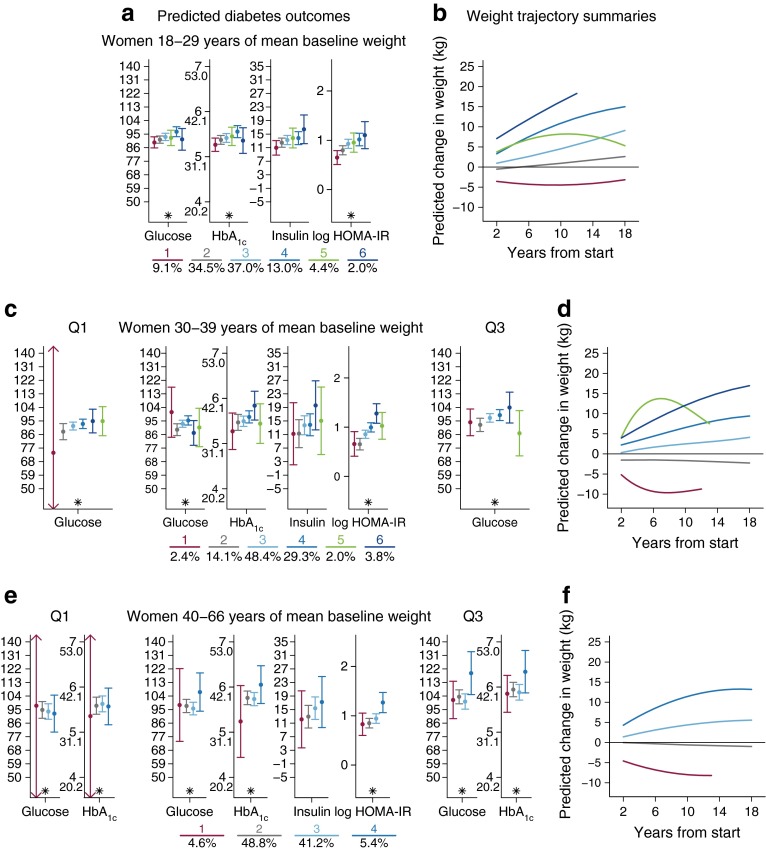
Predicted metabolic markers of diabetes by corresponding weight change trajectory summaries across three age strata in women. Predicted diabetes outcomes were generated from general linear mixed models: (**a**, **b**) 18–29 years; (**c**, **d**) 30–39 years; (**e**, **f**) 40–66 years. Data represent the expected outcomes with 95% CIs at three baseline weights: the sex-specific 25th percentile (quartile Q1), average, and 75th percentile (quartile Q3), where interaction between trajectory class and baseline weight was statistically significant (**c**, **e**), and by average baseline weight where the interaction was not statistically significant (**a**). Each *y*-axis corresponds to the outcome noted below the figure. Results are shown for individuals with a mean baseline weight (54 kg) and mean adult height (156 cm), living in an average community. The percentage sample in each class is shown below the graphs. *p < 0.05 for group differences in an overall test for weight trajectories. Weight trajectory summaries show a different colour for each weight trajectory class (**b**, **d**, **f**), with percentage of sample in each class shown below parts (**a**), (**c**) and (**e**). Shorter lines in (**b**), (**d**) and (**f**) refer to a shorter study period for individuals who entered the study in the mid-2000s

Each participant was assigned a trajectory variable based on the posterior probability of membership in each class: the median assignment probability for assigned classes was 0.70 (interquartile range 0.55–0.92) [8]. The shape of the latent class weight trajectory curves was determined for each age and sex group (Figs 1a, c, e and 2a, c, e; each weight trajectory class is represented by a different colour). The association of each trajectory class with each diabetes-related outcome measure was determined (Figs 1 and 2; parts a, c, e; asterisks indicate statistically significant group differences in weight trajectories). Results are shown for mean baseline weight, except where the interaction between trajectory class variables and baseline weight was statistically significant. Each age–sex stratum included a different set of weight gain trajectory classes.

#### Analysis of men

For men, there was a differential effect of class trajectories by baseline weight for the 18–29 years and 40–66 years age strata, and for glucose only (Fig. 1). Among men aged 18–29 years at baseline (Fig. 1a), there were significant group differences across all outcomes, with higher glucose, HbA_1c_, insulin and log HOMA-IR in the high weight gain (dark blue) trajectory than in all other trajectories. In general, diabetes markers were higher in all weight gain trajectory classes (blue shades) than in the stable weight or low weight gain trajectory class (grey). Statistically significant group differences for all outcomes indicate that glucose, HbA_1c_, insulin and log HOMA-IR varied across trajectory classes.

Although there was a differential baseline weight effect for glucose, the pattern of association was similar across the 25th percentile, mean and 75th percentile of baseline weight. The most significant differences among groups (ESM Tables 2–6; *p* < 0.0001) were observed for HOMA-IR, with considerably higher values observed for the classes with a greater increase in weight over time (groups 3 and 4) relative to those with less or no weight gain (groups 1 and 2).

In men aged 30–39 years (Fig. 1c), there were significant group differences in log HOMA-IR, with higher HOMA-IR values in the weight gain trajectories (blue shades) than in the weight maintenance (grey) and low weight gain (maroon) classes. For men aged 40–66 years at baseline (Fig. 1e), patterns were similar to those for men aged 18–29 years: there were significant group differences across all outcomes except for HbA_1c_, and higher glucose, insulin and log HOMA-IR in the high weight gain (dark blue) group relative to the other trajectories.

There was a differential effect of class trajectories by baseline weight on glucose. Gradations in the levels of cardiometabolic markers were clearest for insulin and log HOMA-IR, with the highest values in the weight gain trajectories (blue shades) and lowest values in the maintenance (grey) and low weight gain (maroon) trajectory classes.

Among men, the pattern of HOMA-IR values was consistent across the three age strata: the higher weight gain trajectories had higher log HOMA-IR values relative to the stable weight or declining weight trajectories. Differences among weight trajectories remained statistically significant even after accounting for baseline weight.

#### Analysis of women

Among women, differential effects of class trajectories by baseline weight were seen for baseline 30–39 years and 40–66 years cohorts, but not for the age 18–29 years baseline cohort. In the 18–29 years baseline cohort (Fig. 2a), glucose, HbA_1c_ and log HOMA-IR were generally higher in the higher weight change trajectories (blue shades and green) than in the other trajectories, despite some overlap in CIs. However, there were some exceptions. The strongest group differences (ESM Tables 5–7; *p* < 0.0001) were observed for HOMA-IR across all age groups, with considerably higher values for groups with increasing weight over time (3, 4, 6 and 5) than in the weight maintenance (2) and low weight gain (1) classes.

For women aged 30–39 years (Fig. 2c), there was a differential effect of baseline weight for glucose, albeit with lower significance at low and average baseline weights. The trajectory of one of the lower weight classes (5 green) was curvilinear, with an initial increase followed by a loss of weight. This class had low glucose and log HOMA-IR values compared with other weight gain classes. Findings for log HOMA-IR were similar to those of the 18–29 years baseline age group: a stepwise increase in log HOMA-IR was observed for the higher weight gain trajectories (light and dark blue). For the cohort aged 40–66 years at baseline (Fig. 2e), significant group differences were seen for all outcomes except insulin, with increasing values for trajectories with increasing weight gain.

There was a differential effect of class trajectories by baseline weight on glucose and HbA_1c_ levels. Gradations in diabetes-related marker levels were clearest for glucose and log HOMA-IR, with highest values in the high weight gain trajectories and lowest values in the weight maintenance and reduction trajectory classes.

### Weight trajectory ranking by metabolic markers of diabetes

Results from our simulation study into the relative ranking of weight trajectories across baseline weight (values for diabetes-related markers: high [1] to low [6]) are shown in ESM Fig. 1. The flat vertical lines show the stability of ranking across baseline weight, while the ‘wavy’ curves for glucose and HbA_1c_ in some age groups indicate differential association across baseline weight categories. For women with baseline age 18–29 years (top row), the class with the highest weight gain trajectory (dark blue) always had the highest marker values, and the class with the second highest weight gain trajectory (medium blue) was often classified into the first or second rank. Results were similar for men: a higher proportion of the high weight gain trajectories were in the highest rank, and a higher proportion of the low weight gain trajectories were in the lowest rank. Across all strata, log HOMA-IR showed the most defined separation of ranks, while HbA_1c_ (with the exception of men aged 18–29 years) was generally quite mixed.

A sensitivity analysis in which we excluded 139 people who reported taking diabetes medication or insulin, or who reported being diagnosed with diabetes by a doctor (ESM Figs 2 and 3), showed substantial similarity in point estimates, albeit with slightly larger CIs.

## Discussion

Our analysis of 18 year data from China suggests that metabolic markers of diabetes and insulin resistance vary across different trajectories of weight change. For the most part, these differences were independent of baseline weight, with the exception of glucose for some of the baseline age groups. Levels of glucose, insulin and HOMA-IR were elevated across all weight gain groups relative to weight maintainers, especially in individuals with steeper weight trajectories through adulthood. This was true even after adjusting for initial weight, height and smoking, and accounting for the community of residence. Our findings reveal variations in the values of diabetes-related markers across differential patterns of weight gain that are masked in an unstratified classification of weight gain. These patterns are worrying because more rapid weight gain has recently become common in China, and children are now entering their adult years at higher body weights than in previous generations. Currently, diabetes accounts for approximately 80% of deaths in low- and middle-income countries [34], and China has experienced a rapid increase in diabetes and other cardiometabolic diseases [18, 35, 36].

We observed more group differences in the values of diabetes-related markers in strata comprising younger and older men (18–29 years and 40–66 years) than in the middle stratum (30–39 years). An exception was HOMA-IR, which showed group differences across all ages. For women, the two younger strata (18–29 years and 30–39 years) had more group differences than the older stratum (40–66 years), except for HOMA-IR, which again showed group differences across all ages. Marker values were lower for classes that maintained weight, lost weight or switched from weight gain to weight loss; however, some nonstatistically significant differences might relate to the smaller group sizes of some trajectory classes. Nonetheless, our findings highlight a critical need for preventive strategies to reduce weight gain in early adulthood to avoid diabetes and insulin resistance risk.

Given that insulin resistance and diabetes correlate highly with current BMI, we examined associations in models with our derived trajectories and baseline weight (which effectively describe an individual’s current weight contemporaneous with diabetes-related markers) relative to the models using baseline weight only. Our finding of different associations in these two sets of models suggests that the shape of the trajectories over time (i.e. weight history) is linked to differential values for markers of diabetes and insulin resistance.

While other studies have shown that duration of obesity is associated with the occurrence of cardiometabolic risk factors [37–39], few have examined weight trajectories. However, studies using latent trajectory methods across the lifespan have shown an association between differential weight trajectories and cardiometabolic risk factors or mortality [11, 40–43]. LCTA has only recently been used to identify patterns in epidemiological data [14, 29, 30, 44, 45]. Our use of LCTA for the flexible modelling of weight patterns in this study indicates heterogeneity in weight trajectories across diabetes outcomes. In particular, we found stronger associations with a trajectory of higher initial weight gain that is maintained over the entire lifespan compared with groups exhibiting a recent, rapid increase in weight. While other studies have used principal components analysis to characterise patterns of weight change related to diabetes risk [6, 7], our LCTA approach provides a more detailed examination of differential weight change trajectories and diabetes-related outcomes. In contrast to approaches that use population averaging, our findings suggest that identifying subgroups with differential patterns of weight gain is useful for classifying levels of diabetes-related markers.

There are a few limitations to our analysis. We examined the association between weight trajectories and higher glucose, HbA_1c_, HOMA-IR and insulin levels in 2009, but we do not know when elevations in these diabetes markers first developed. Our main objective was to examine the association between 18 year weight trajectories and diabetes- and insulin-related outcomes. The analysis strategy was therefore designed to adjust for key covariates, rather than for causal modelling of these relationships. We could not distinguish type 1 from type 2 diabetes, although type 1 diabetes incidence in China is among the lowest in the world, at an estimated 0.1 per 100,000 per year [46]. Given our inability to remove individuals with undiagnosed diabetes, which occurs at a very high rate in China [47–49], we did not exclude the 139 individuals who reported taking diabetes medications or insulin, or who had been diagnosed with diabetes by a doctor (findings are summarised in ESM Figs 1 and 2). We examined weight trajectories to inform large-scale, population-based preventive efforts, although we realise that visceral and liver fat also play roles in the aetiology of diabetes and insulin resistance. Despite our large overall study population, our examination of differences in age and sex strata resulted in small numbers in some trajectory classes, which limited the power to detect significant differences in some groups. Although multiple comparisons are an issue when comparing trajectory classes across sex and age strata, we have more confidence in patterns that were repeated across sex or age classes, even if they were not statistically significant. Finally, our inclusion criteria resulted in the selection of a subset of the total study sample. The study population was younger, more urban and less likely to smoke than the total population, and these characteristics could have influenced patterns of weight gain and increased the values of diabetes and insulin resistance markers.

## Conclusion

Diabetes has become a major public health concern across China. While obesity is a major risk factor for diabetes, it is less well understood how weight gain trajectories relate to diabetes and insulin resistance. Using latent class trajectory models to identify distinct groups with similar underlying patterns of longitudinal weight change, we found lower levels of diabetes markers in classes that maintained weight, lost weight or switched from weight gain to weight loss. More group differences were seen in younger age groups. These findings highlight the importance of reducing weight gain in early adulthood to reduce diabetes risk.

## Electronic supplementary material

Below is the link to the electronic supplementary material.

ESM Table 1 (PDF 59 kb)

ESM Table 2 (PDF 44.9 kb)

ESM Table 3 (PDF 43.1 kb)

ESM Table 4 (PDF 46 kb)

ESM Table 5 (PDF 44.2 kb)

ESM Table 6 (PDF 48.9 kb)

ESM Table 7 (PDF 47 kb)

ESM Fig. 1 (PDF 7887 kb)

ESM Fig. 2 (PDF 125 kb)

ESM Fig. 3 (PDF 118 kb)

## Abbreviations

CHNS: China Health and Nutrition Survey
LCTA: Latent class trajectory analysis
