# Supplementary material for: Eighteen year weight trajectories and metabolic markers of diabetes in modernising China
Source: Diabetologia. 2014 Jun 3;57(9):1820–9. doi: 10.1007/s00125-014-3284-y (PMC4119243; doi:10.1007/s00125-014-3284-y)
Supplement: Supplementary file 1 — (PDF 59 kb) [file 125_2014_3284_MOESM1_ESM.pdf]

ESM Table 1. Missingness for the Analytic Sample			
	**Included**	**Excluded**<br>**(subjects with at least one adult measurement less than 66 in 2009 with no trajectory or no fasting, or pregnant)**	***p*-value**
	**n=5436**	**n=2713**	
2009 Age	49.0 (40.8 -- 56.8)	39.0 (27.0 -- 51.3)	<0.0001
Baseline Weight	56.4 (51.0 -- 63.5)	58.9 (51.9 -- 66.0)	<0.0001
Mean Adult Height	160.9 (155.7 -- 167.0)	162.2 (157.0 -- 169.0)	<0.0001
Total Household Income	32489 (17902 -- 55703)	32190 (18911 -- 52632)	0.6756
2009 Urbanisation Index	61.0 (50.6 -- 83.0)	74.7 (53.9 -- 89.1)	<0.0001
Ever Smoke			0.0033
0	68.0% (3696)	43.7% (1185)	
1	31.9% (1736)	17.1% (463)	
Missing	0.1% (4)	39.3% (1065)	
