# Supplementary material for: Eighteen year weight trajectories and metabolic markers of diabetes in modernising China
Source: Diabetologia. 2014 Jun 3;57(9):1820–9. doi: 10.1007/s00125-014-3284-y (PMC4119243; doi:10.1007/s00125-014-3284-y)
Supplement: Supplementary file 2 — (PDF 44.9 kb) [file 125_2014_3284_MOESM2_ESM.pdf]

ESM Table 2. Summary of Results for Males Baseline Age 18 to 29 years						
**Outcome**	**Trajectory**	**Difference from Sex Specific Mean Baseline Weight**	**n**	**Interaction *p*-value**	**Overall *p*-value**	**Group Differences**
Glucose	1	-7	80	0.0201	0.0005	4
	2	-7	523			3 4
	3	-7	248			2
	4	-7	41			1 2
	1	0	80			3 4
	2	0	523			3 4
	3	0	248			1 2 4
	4	0	41			1 2 3
	1	5	80			4
	2	5	523			
	3	5	248			
	4	5	41			1
HbA_{1c}	1	0	79	0.1153	0.0046	4
	2	0	520			3 4
	3	0	247			2 4
	4	0	40			1 2 3
Insulin	1	0	80	0.2016	<0.0001	
	2	0	524			3 4
	3	0	248			1 2
	4	0	40			1 2
log HOMA-IR	1	0	80	0.1953	<0.0001	
	2	0	523			1 3 4
	3	0	248			1 2
	4	0	40			1 2
