# Supplementary material for: Eighteen year weight trajectories and metabolic markers of diabetes in modernising China
Source: Diabetologia. 2014 Jun 3;57(9):1820–9. doi: 10.1007/s00125-014-3284-y (PMC4119243; doi:10.1007/s00125-014-3284-y)
Supplement: Supplementary file 3 — (PDF 43.1 kb) [file 125_2014_3284_MOESM3_ESM.pdf]

ESM Table 3. Summary of Results for Males Baseline Age 30 to 39 years						
**Outcome**	**Trajectory**	**Difference from Sex Specific Mean Baseline Weight**	**n**	**Interaction *p*-value**	**Overall *p*-value**	**Group Differences**
Glucose	1	0	44	0.421	0.3209	
	3	0	300			
	2	0	352			
	4	0	120			
	5	0	16			
HbA_{1c}	1	0	44	0.0666	0.3644	
	3	0	298			
	2	0	343			
	4	0	120			
	5	0	16			
Insulin	1	0	44	0.8282	0.7758	
	3	0	298			
	2	0	351			
	4	0	119			
	5	0	16			
log HOMA-IR	1	0	44	0.5303	<0.0001	
	3	0	298			2 4 5
	2	0	350			1 3 4 5
	4	0	119			1 2 3
	5	0	16			1 2 3
