# Supplementary material for: Eighteen year weight trajectories and metabolic markers of diabetes in modernising China
Source: Diabetologia. 2014 Jun 3;57(9):1820–9. doi: 10.1007/s00125-014-3284-y (PMC4119243; doi:10.1007/s00125-014-3284-y)
Supplement: Supplementary file 4 — (PDF 46 kb) [file 125_2014_3284_MOESM4_ESM.pdf]

ESM Table 4. Summary of Results for Males Baseline Age 40 to 66y						
**Outcome**	**Trajectory**	**Difference from Sex Specific Mean Baseline Weight**	**n**	**Interaction *p*-value**	**Overall *p*-value**	**Group Differences**
Glucose	1	-7	27	0.0282	0.0477	
	2	-7	258			
	3	-7	384			
	4	-7	139			
	5	-7	41			
	1	0	27			
	2	0	258			
	3	0	384			
	4	0	139			
	5	0	41			
	1	5	27			
	2	5	258			
	3	5	384			
	4	5	139			
	5	5	41			
HbA_{1c}	1	0	27	0.0617	0.7882	
	2	0	258			
	3	0	384			
	4	0	139			
	5	0	40			
Insulin	1	0	27	0.8689	0.0132	4 5
	2	0	258			3 4 5
	3	0	383			2
	4	0	139			1 2
	5	0	40			1 2
log HOMA-IR	1	0	27	0.4133	<0.0001	
	2	0	258			4 5
	3	0	383			1 4 5
	4	0	139			1 2 3
	5	0	40			1 2 3
