# Supplementary material for: Eighteen year weight trajectories and metabolic markers of diabetes in modernising China
Source: Diabetologia. 2014 Jun 3;57(9):1820–9. doi: 10.1007/s00125-014-3284-y (PMC4119243; doi:10.1007/s00125-014-3284-y)
Supplement: Supplementary file 5 — (PDF 44.2 kb) [file 125_2014_3284_MOESM5_ESM.pdf]

ESM Table 5. Summary of Results for Females Baseline Age 18 to 29 years						
**Outcome**	**Trajectory**	**Difference from Sex Specific Mean Baseline Weight**	**n**	**Interaction *p*-value**	**Overall *p*-value**	**Group Differences**
Glucose	1	0	81	0.6287	0.0162	4
	2	0	307			4
	3	0	333			4
	5	0	39			
	4	0	117			1 2 3
	6	0	18			
HbA_{1c}	1	0	81	0.5486	0.0247	3 4
	2	0	308			4
	3	0	329			1 4
	5	0	39			
	4	0	115			1 2 3
	6	0	18			
Insulin	1	0	81	0.4799	0.0999	
	2	0	308			
	3	0	333			
	5	0	40			
	4	0	117			
	6	0	18			
log HOMA-IR	1	0	81	0.2609	<0.0001	
	2	0	306			1 3 4 6
	3	0	333			1 2
	5	0	39			1
	4	0	117			1 2
	6	0	18			1 2
