# Supplementary material for: Eighteen year weight trajectories and metabolic markers of diabetes in modernising China
Source: Diabetologia. 2014 Jun 3;57(9):1820–9. doi: 10.1007/s00125-014-3284-y (PMC4119243; doi:10.1007/s00125-014-3284-y)
Supplement: Supplementary file 6 — (PDF 48.9 kb) [file 125_2014_3284_MOESM6_ESM.pdf]

ESM Table 6. Summary of Results for Females Baseline Age 30 to 39 years						
**Outcome**	**Trajectory**	**Difference from Sex Specific Mean Baseline Weight**	**n**	**Interaction *p*-value**	**Overall *p*-value**	**Group Differences**
Glucose	1	-5.5	25	0.0053	0.0004	
	2	-5.5	148			
	3	-5.5	509			
	4	-5.5	308			
	6	-5.5	40			
	5	-5.5	21			
	1	0	25			
	2	0	148			4
	3	0	509			
	4	0	308			2
	6	0	40			
	5	0	21			
	1	6	25			
	2	6	148			4 6
	3	6	509			
	4	6	308			2
	6	6	40			2
	5	6	21			
HbA_{1c}	1	0	25	0.3547	0.1754	
	2	0	148			
	3	0	509			
	4	0	307			
	6	0	40			
	5	0	21			
Insulin	1	0	25	0.9999	0.4655	
	2	0	149			
	3	0	510			
	4	0	308			
	6	0	40			
	5	0	21			
log HOMA-IR	1	0	25	0.7696	<0.0001	
	2	0	148			3 4 5 6
	3	0	508			2 4 6
	4	0	308			1 2 3 6
	6	0	40			1 2 3 4
	5	0	21			1 2
