# Supplementary material for: Eighteen year weight trajectories and metabolic markers of diabetes in modernising China
Source: Diabetologia. 2014 Jun 3;57(9):1820–9. doi: 10.1007/s00125-014-3284-y (PMC4119243; doi:10.1007/s00125-014-3284-y)
Supplement: Supplementary file 7 — (PDF 47 kb) [file 125_2014_3284_MOESM7_ESM.pdf]

ESM Table 7. Summary of Results for Females Baseline age 40 to 66 years						
**Outcome**	**Trajectory**	**Difference from Sex Specific Mean Baseline Weight**	**n**	**Interaction *p*-value**	**Overall *p*-value**	**Group Differences**
Glucose	1	-5.5	41	0.0415	0.0161	
	2	-5.5	434			
	3	-5.5	365			
	4	-5.5	48			
	1	0	41			
	2	0	434			
	3	0	365			
	4	0	48			
	1	6	41			
	2	6	434			4
	3	6	365			4
	4	6	48			2 3
HbA_{1c}	1	-5.5	41	0.0027	0.0027	
	2	-5.5	432			
	3	-5.5	365			
	4	-5.5	48			
	1	0	41			
	2	0	432			
	3	0	365			
	4	0	48			
	1	6	41			
	2	6	432			
	3	6	365			
	4	6	48			
Insulin	1	0	41	0.7394	0.4397	
	2	0	433			
	3	0	366			
	4	0	48			
log HOMA-IR	1	0	41	0.2331	0.0007	4
	2	0	433			4
	3	0	365			4
	4	0	48			1 2 3
