# Supplementary material for: Eighteen year weight trajectories and metabolic markers of diabetes in modernising China
Source: Diabetologia. 2014 Jun 3;57(9):1820–9. doi: 10.1007/s00125-014-3284-y (PMC4119243; doi:10.1007/s00125-014-3284-y)
Supplement: Supplementary file 8 — (PDF 7887 kb) [file 125_2014_3284_MOESM8_ESM.pdf]

ESM Figure 1. Relative rank of weight trajectories across baseline weight for diabetes−related risk across sex and age strata, results from a simulation study.

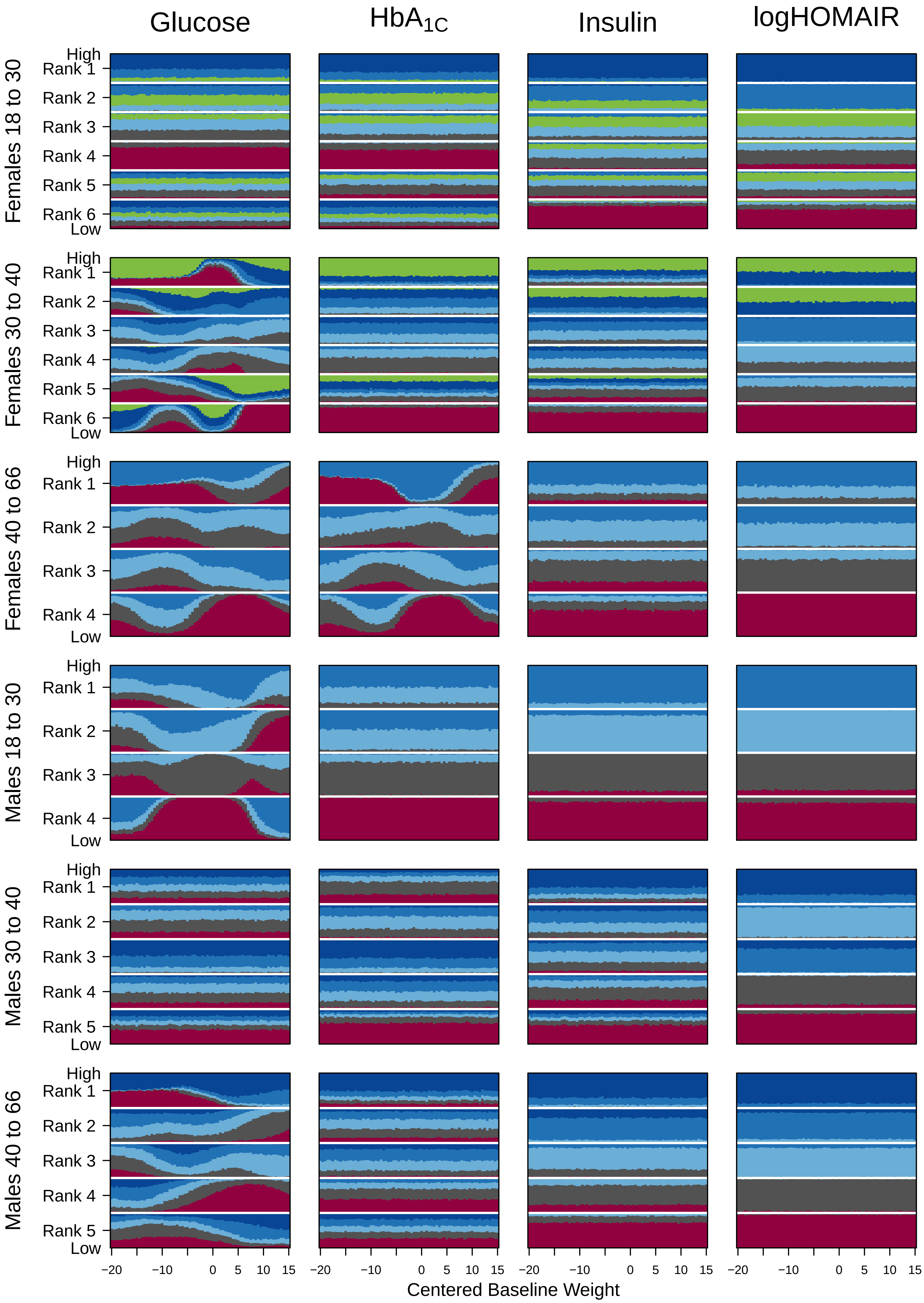


Results from a simulation based on random sampling from normal distributions for each trajectory class in each age−sex strata across a range of baseline weight, centered at the trajectory−specific estimates of each outcome and their respective variances at the particular baseline weight. Ranks of the trajectory groups were recorded with rank 1 given to the trajectory class with the highest estimate for each outcome measure. This process was repeated 1000 times and the frequency of each ranking shown with color coding matching the trajectory figures in Figures 1 and 2, according to the frequency that each age−sex−specific trajectory class was assigned a particular rank ranging from low values for each outcome measure to high values of each outcome measure. Note that flat vertical lines show stability of rank across baseline weight, while the wavy curves indicate differential association across baseline weight categories.
