# Supplementary material for: Eighteen year weight trajectories and metabolic markers of diabetes in modernising China
Source: Diabetologia. 2014 Jun 3;57(9):1820–9. doi: 10.1007/s00125-014-3284-y (PMC4119243; doi:10.1007/s00125-014-3284-y)
Supplement: Supplementary file 10 — (PDF 118 kb) [file 125_2014_3284_MOESM10_ESM.pdf]

ESM Figure 3. Predicted metabolic markers of diabetes (left panel) by corresponding weight change trajectory summaries (right panel) across three age strata for women, without 139 people who reported taking diabetes medications, insulin, or reported that they had been diagnosed with diabetes by a doctor.

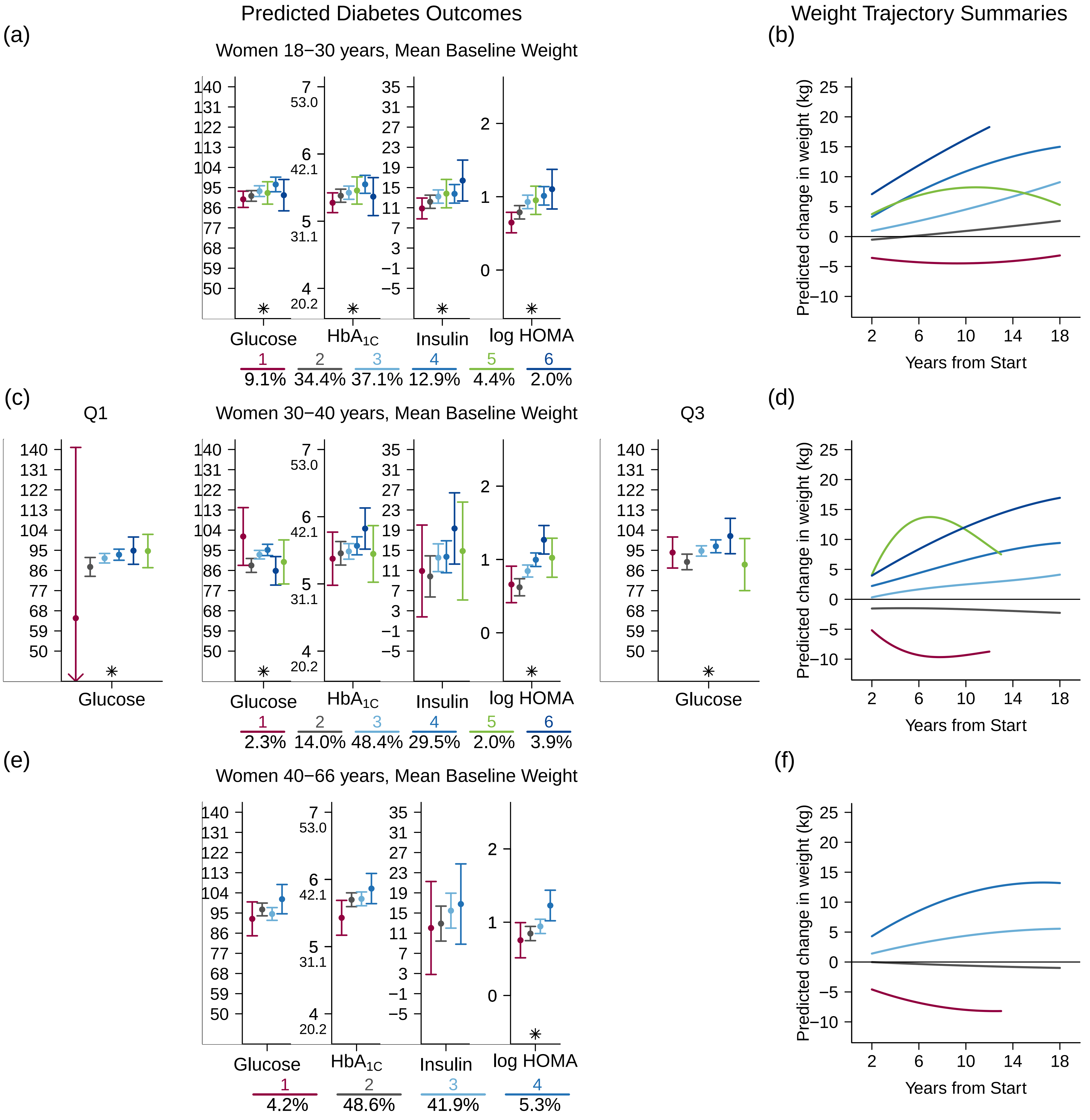

Predicted diabetes outcomes are generated from general linear mixed models (a) 18−30 years; (c) 30−40 years; (e) presented as expected outcomes with 95% confidence intervals at three baseline weights: the gender−specific 25th percentile [Quartile (Q1)], average, and 75th percentile [Quartile (Q3)], where interaction between trajectory class and baseline weight was statistically significant (c) and by average baseline weight where the interaction was not statistically significant (a) and (e). Results shown for women with mean baseline weight (54 kg) and mean adult height (156 cm), living in an average community. Asterisks indicate statistically significant group differences in an overall test for weight trajectories. Weight trajectory summaries are shown in (b), (d), and (f) with separate colors indicatingeach weight trajectory class, percentage of sample in each class is shown in the index. Note that shorter lines refer to shorter length of time in study for individuals who entered the study in the mid−2000's.
